# Design of Heat Exchanger for Ericsson-Brayton Piston Engine

**DOI:** 10.1155/2014/138254

**Published:** 2014-05-28

**Authors:** Peter Durcansky, Stefan Papucik, Jozef Jandacka, Michal Holubcik, Radovan Nosek

**Affiliations:** University of Zilina, Univerzitná 8215/1, 010 26 Zilina, Slovakia

## Abstract

Combined power generation or cogeneration is a highly effective technology that produces heat and electricity in one device more efficiently than separate production. Overall effectiveness is growing by use of combined technologies of energy extraction, taking heat from flue gases and coolants of machines. Another problem is the dependence of such devices on fossil fuels as fuel. For the combustion turbine is mostly used as fuel natural gas, kerosene and as fuel for heating power plants is mostly used coal. It is therefore necessary to seek for compensation today, which confirms the assumption in the future. At first glance, the obvious efforts are to restrict the use of largely oil and change the type of energy used in transport. Another significant change is the increase in renewable energy—energy that is produced from renewable sources. Among machines gaining energy by unconventional way belong mainly the steam engine, Stirling engine, and Ericsson engine. In these machines, the energy is obtained by external combustion and engine performs work in a medium that receives and transmits energy from combustion or flue gases indirectly. The paper deals with the principle of hot-air engines, and their use in combined heat and electricity production from biomass and with heat exchangers as primary energy transforming element.

## 1. Introduction

**Figure 1:**
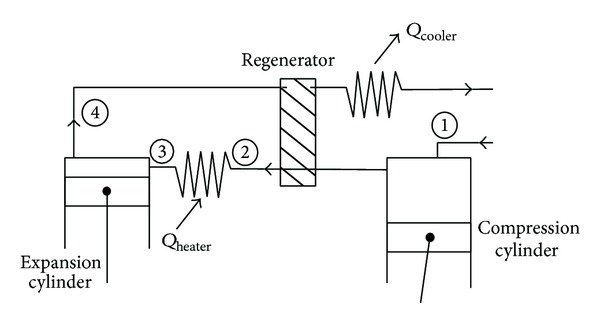
Scheme of Ericsson-Brayton hot-air engine with open cycle.

*Microcogeneration Unit with Nonconventional Engine*. Combustion engines with gas as fuel, with the mostly used being natural gas, are most used as power unit of microcogeneration devices. Losses in electricity generation are mainly associated with imperfect energy transformation in burning fuel in an imperfect transformation of energy working medium in the turbine. There are also represented mechanical losses and loss of energy in transmission lines. The minimal losses have cogeneration plants. Cogeneration unit is a technical device, which is manufactured by electric and thermal energy simultaneously. As an example cogeneration unit may be mentioned with an internal combustion gas engine. The engine burns the gas, thereby gaining the mechanical power on the shaft to drive an electric generator. The engine has no classic cooler but has the heat exchanger from which we obtain thermal energy. Used heat exchangers are connected in series circuits, where the working medium, usually water, is preheated and heated in several stages. Overall efficiency of CHP unit can be increased with multistage heat recovery and this reduces also the total cost of fuel [1]. Unconventional engines are a possible alternative to the internal combustion engines. They work with external combustion, which allows, unlike conventional internal combustion engines, controlling the course of combustion and therefore its quality, which is reflected in the composition of air pollutants emitted to the atmosphere. The most known hot-air engines are Stirling and Ericsson engines. Ericsson engine is also an external combustion engine. In contrast to Stirling engine, it has two possible alternatives—open and closed [2]. In the case of Stirling engine dual function of regenerator is immediately apparent. Regenerator works as heater and cooler while in Ericsson engine cooler and heater are separated.  Figure 1 presents Ericsson-Brayton engine with open cycle.

The air is compressed in the compressor and flows through the heat exchanger, where at constant pressure it is receiving heat. Consequently, it is led to the expansion cylinder, which expands adiabatically. Part of this work will be used to drive the compressor and part is used as mechanical work to drive an electric generator. As the heat source can be used almost any fuel for burning, as it is an external combustion engine. Fuel is burned in a separate combustion chamber and heat energy is transformed through a heat exchanger to the working media. The working medium in open cycle, mostly dry air, after passing the cycle, is discharged into the atmosphere. In a closed cycle the medium after each cycle cools in refrigerant heat exchanger, where it gives heat energy and is fed back into the cycle. With the use of closed cycle we can improve the efficiency of heating equipment [3, 4].

**Figure 2:**
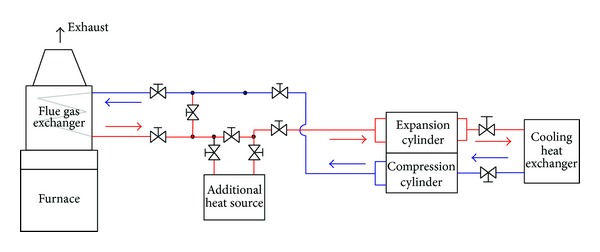
Experimental scheme of Ericsson-Brayton hot-air engine with open cycle.

The proposed microcogeneration unit uses two heat exchangers: cooler and heater (see Figure 2). A different purpose sets other requirements for the heat exchangers. The first requirement is to ensure optimal heat transfer between flowing media. The heat transfer is characterized by a heat transfer coefficient. This summary represents the characteristics of the heat exchanger, its layout, and the flowing media. Coefficient depends on the characteristics of the flowing media, from the heat capacity, and the selected construction option and in some cases is significantly influenced by the material used and the heat exchanger. The requirement is that the coefficient is the highest while respecting the chosen solutions. Further requirements are then asked to compact size exchanger and the total pressure loss and also maintenance options are required [3, 4].

## 2. Heat Exchanger Design 

As the first step, the working conditions of the CHP were set. The experimental application with Ericsson-Brayton hot-air engine sets a wide range of specifications, not only on the heat exchanger, but also on the whole system. The whole unit should supply energy for household. In the determination of the operating conditions we have preliminary set the highest temperatures from 500°C up to 620°C, according to [3, 5]. In this paper, the authors presented the highest temperature of 600°C. Mr. Creyx [1] has presented systems with different working fluids and also different hot-air engine configurations. The system presented in this paper should work with closed cycle, with dry air as working fluid [4]. The closed cycle enables heat recovery from working fluid, so the regenerated heating power is bigger than that in opened cycle, where the most part of heat energy is used to preheat the air after compression. It is assumed that temperature of the working fluid after expansion is in the range of 240°C–320°C [4, 5]. For each working fluid, the dry air in the tubes and the exhaust gases outside the tubes were set as the characteristic temperatures and physical properties.

There are many ways for how to calculate the properties of flowing media. In order to determine the heat transfer, it was necessary to know the thermodynamic properties of flowing gas. It is important to determine the dynamic and kinematic viscosity. For heat transfer the thermal conductivity of the gas is also needed to be known. The following equations were used for calculation [6, 7].

Dynamic viscosity is

(1) $$\begin{array}{c} \eta_{TP}=1.0607\cdot 10^{-6}\cdot T^{0,5}\cdot k_{T}\cdot k_{p}. \end{array}$$

Kinematic viscosity is

(2) $$\begin{array}{c} \nu_{TP}=304.52344\cdot 10^{-6}\frac{T^{1,5}}{p}k_{T}k_{P}, \end{array}$$

and the thermal conductivity is

(3) $$\begin{array}{c} \lambda =1513.8151\cdot 10^{-6}T^{0,5}{\left(k_{T}k_{P}\right)}^{1,5}. \end{array}$$

The coefficients *k*_*T*_ and *k*_*p*_ are based on temperatures from 0°C up to 1000°C. The main difference to real values of parameters is up to 3%, so it is possible to say that the computation is accurate.  Table 1 shows the values of coefficient *k*_*T*_.

The values of the coefficient *k*_*P*_ are set for dry air by constant temperature. In Table 2, some values of coefficient *k*_*P*_ can be seen.

There are many methods for calculation of flue gas density. Two of them were used in this work. At first it is possible to read the right values in the tables, which are calculated or measured. In Table 3 there are some values of air properties. The second column is density. The values that are not in the table can be calculated.

Density can be calculated based on known parameters. In the following equation density is expressed as a function of dynamic and kinematic viscosity:

(4) $$\begin{array}{c} \rho =\frac{\mu }{\nu }. \end{array}$$

So in this way we can define the properties of flowing medium. Specifying the geometrical properties or features of the chosen type of heat exchanger is also very important. There are many basic concepts of heat exchangers. Based on the geometrical features or heat transfer, methods can be classified into many classes. For the purpose of this work, pipe exchanger was selected. The heat exchanger in this category differs in the arrangement of tubes. The tubes can be organized straight or staggered or partly staggered. It is characterized with the dimensionless constants “*a*” and “*b*.”

**Figure 3:**
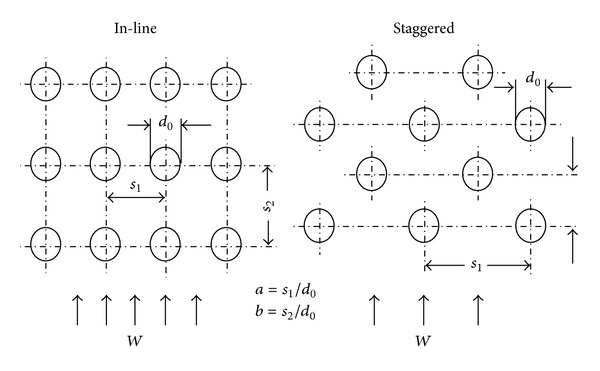
Lateral and longitudinal spacing in tube bundles.

If the tube bundle has horizontal spacing “*s*_1_” and vertical spacing “*s*_2_,” as in Figure 3, the bundle can be characterized with these constants:

(5) a=s1d0  b=s2d0,ψ=1−π4·a.

The streamed length “*l*” can be expressed as length of flow path across over a single tube [7]:

(6) $$\begin{array}{c} l=\frac{\pi }{2}\cdot d_{o}. \end{array}$$

Another difference is in the nondimensional criteria. Reynolds number is characterizing the flowing medium and the type of flow. It depends on flow velocity and also on the geometry. For heat transfer through tubes in bundle the following Reynolds number criteria were used:

(7) Re=w·lψ·ν.

Nusselt number is characterizing the heat transfer. If the turbulence in the inflowing medium is low, deviations in the Nusselt number may occur. The average Nusselt number in a cross-flow over a bundle of smooth tubes can be calculated from that in a cross-flow over a single tube. For the purpose of this work, the criteria equation was used according to [7, 8]. The heat transfer is described by the 2 parts of flow, the turbulent part and the laminar part of the flow near the walls, as follows:

(8) Nul,lam=0.664·Reψ,l·Pr3,Nul,turb.=0.037·Reψ,l0,8·Pr1+2.443·Reψ,l−0,1·(Pr2/3−1).

Turbulent flow in pipe sets in at *Re* > 10^4^. In the transition region of Reynolds number from 2300 to 10^4^ the type of flow is also influenced by the nature of inlet stream and the form of pipe inlet. Tube bundles with in-line tubes behave more like parallel channels, which are formed by the tube rows. An expected increase in heat transfer coefficient due to the turbulence enhancement caused by the tube rows does not occur [7].

The application for hot-air Ericsson-Brayton engine will use as primary heat exchanger tube with staggered tubes.

The average Nusselt number for this type of heat transfer through tube bundle is defined according to the following equation [7]:

(9) $$\begin{array}{c} {\text{Nu}}_{0,\text{bundle}}=\frac{1+\left(n-1\right)\cdot fa}{n}\cdot {\text{Nu}}_{l,0}, \end{array}$$

where

(10) fa,stag=1+23b,Nul,0=0.3+Nul,lam2+Nul,turb  2.

Then followed the estimation of overall coefficient of heat transfer, which is depending on the Nusselt number, is

(11) $$\begin{array}{c} \alpha =\;\frac{{\text{Nu}}_{\text{bundle}}\cdot \lambda_{TM}}{l}. \end{array}$$

**Figure 4:**
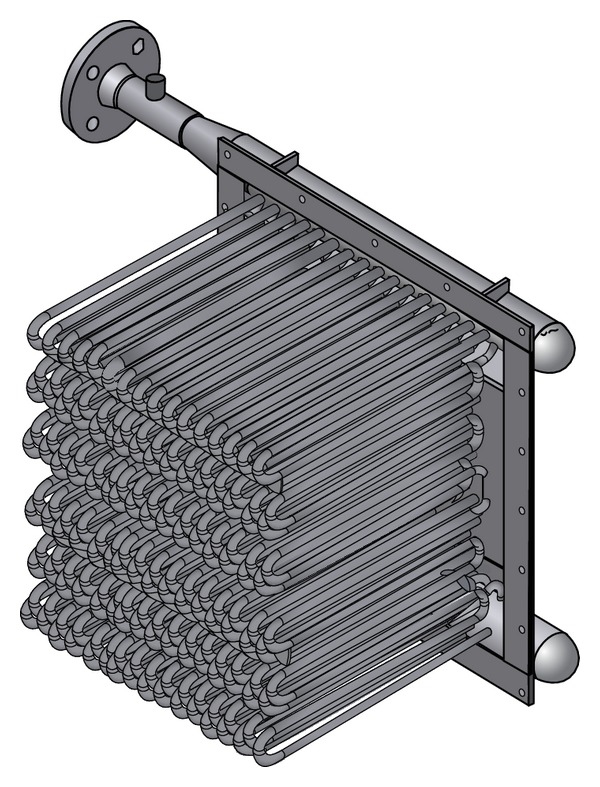
3D model of proposed heat exchanger.

When both sides of equation are known, the overall heat transfer coefficient and the required heat transfer surface can be estimated. Subsequently, 3D model of heat exchanger was created. The model was in the first step created with wall thickness of tubes and inlet tube. But this solution sets major requirements for computing hardware, so a simplified model with tubes as full material was created, the proposal is in Figure 4.

## 3. Heat Exchanger Verification Using Ansys Fluent

The model for Ansys Fluent was created using 3D modeling software. By the creation of the model the substitution of all the construction elements by simple geometrical features was very important [8].

This means that the whole exchanger was created as one volume with tubes as full material. The tubes have multiple collectors at inlet and outflow. No construction tolerances are reflected.

**Figure 5:**
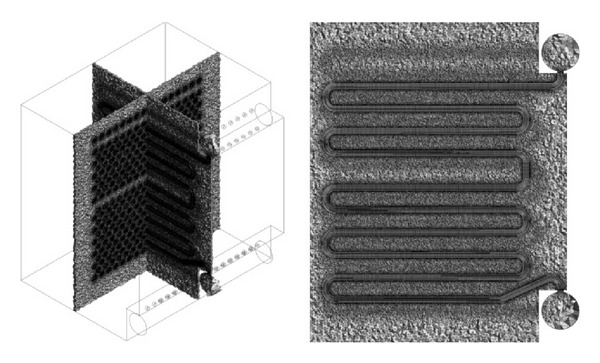
Generated tetrahedral mesh.

**Figure 6:**
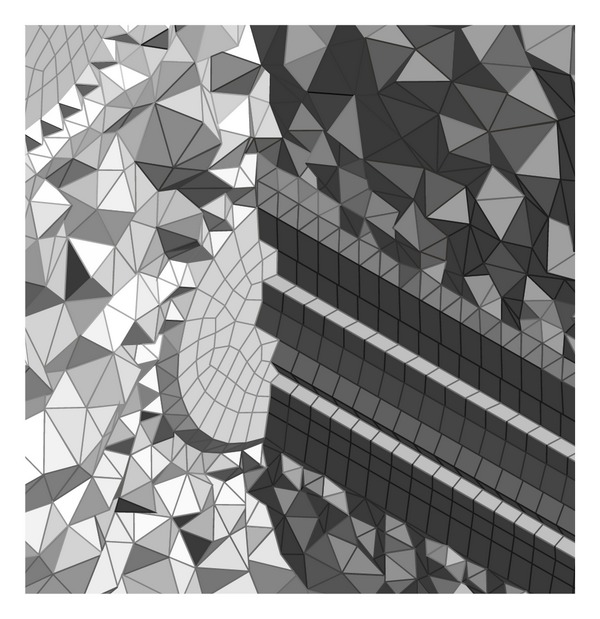
Details of polyhedral mesh.

**Figure 7:**
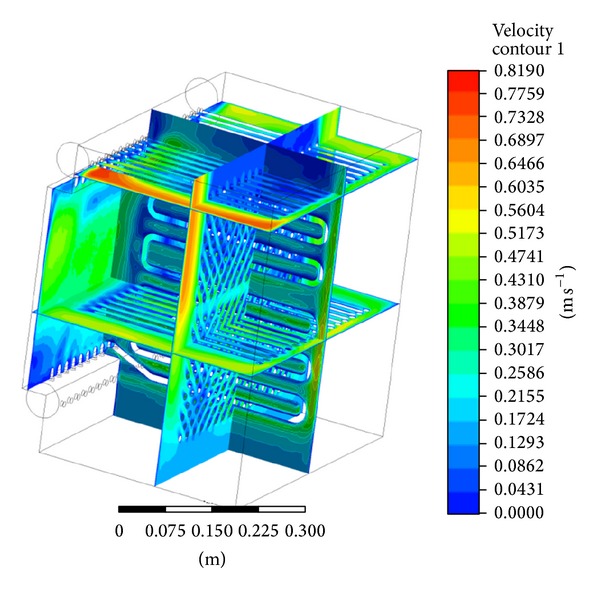
Velocity contour in the proposed heat exchanger.

**Figure 8:**
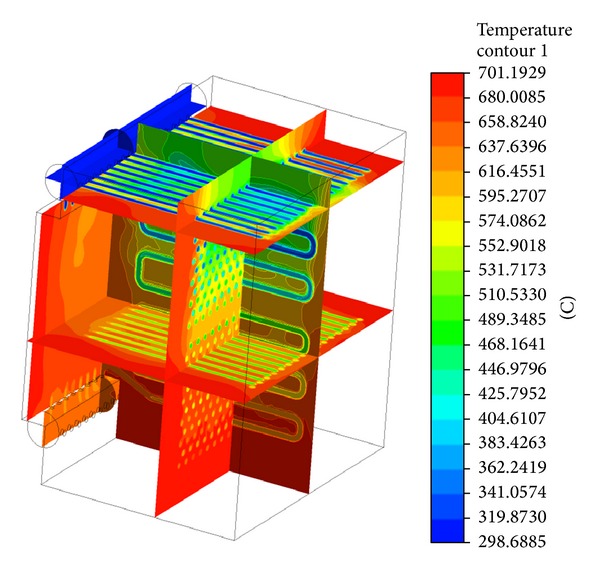
Temperature contour in the proposed heat exchanger.

The exterior of the heat exchanger was created by cutting out the material from volume. In the first step, the tetrahedron mesh was used to fill the whole volume. The generated mesh is displayed in Figure 5. Quality of the generated mesh is determined by skewness of elements and by minimal orthogonal quality. Skewness of the model was 7.1833·10^−7^, where lower value is representing worse quality [8]. Based on this, the tetrahedron mesh was converted to polyhedral mesh. The details of the generated mesh are in Figure 6. The model was solved with polyhedral mesh and *K*-*ε* model. The flow was predicted as turbulent. In Figure 7 the velocity contour is shown and in Figure 8 the temperature field can be observed. The current model has confirmed the mathematical model and also accuracy of chosen geometry.

## 4. Conclusion

Hot-air Ericsson-Brayton engine used in cogeneration unit is a nonconventional engine, which produces electric energy by using different types of fuel, for example, biomass, wood pellets, and so forth. Heat exchanger design for hot-air Ericsson-Brayton engine sets a wide range of specifications. At the first step, the working conditions of whole unit and the required power and temperatures for each part of this device were defined. The basic dimensions of heat exchanger were set using criterion formula. With this calculation, the inlet and outlet temperatures of the heat exchanger were verified. Then, the calculation using Ansys Fluent has followed. As the next step was to complete the construction documentation and finish all design fundamentals, the construction and real measurements can follow.

## Figures and Tables

**Table 1:** Coefficient *k*_*T*_ for dry air by pressure 10^5^ Pa.

*T* [K]	*t* [°C]	*k*_*T*_
373,15	100	1,054403
393,15	120	1,066696
413,15	140	1,075804
433,15	160	1,087817
453,15	180	1,103398

**Table 2:** Coefficient *k*_*p*_ for dry air by temperature 273 K.

*p* [Pa]	*p* [bar]	*k*_*p*_
10	10^−4^	0,464348
10^2^	10^−3^	0,880435
10^3^	10^−2^	0,984783
10^4^	10^−1^	0,993333
10^5^	1	1,000000
5 · 10^5^	5	1,003509

**Table 3:** Physical properties for dry air by pressure 100 kPa.

*t* [°C]	*ρ* [kg/m^3^]	*c* [J/kg·K]	*λ* · 10^2^ [W/(m·K)]	*a* · 10^6^ [m^2^/s]
0	1,275	1005	2,37	18,5
10	1,23	1005	2,45	19,82
20	1,188	1010	2,52	21
40	1,112	1013	2,65	23,53
60	1,046	1017	2,8	26,32
80	0,986	1020	2,93	29,13
100	0,934	1022	3,07	32,16
120	0,886	1024	3,2	35,27
140	0,843	1027	3,33	38,46
160	0,804	1030	3,44	41,54
180	0,769	1034	3,57	44,9
200	0,736	1037	3,7	48,48
